# Reviewer acknowledgement 2013

**DOI:** 10.1186/1868-7083-5-4

**Published:** 2013-04-02

**Authors:** Ulrich Mahlknecht

**Affiliations:** 1 José Carreras Center for Immunotherapy and Gene Therapy, Saarland University Medical Center, Homburg, Saar, Germany

## Abstract

**Contributing reviewers:**

The editors of Clinical Epigenetics would like to thank all our reviewers who have contributed to the journal in volume 4 (2012).

## 

Barry Iacopetta

Australia

Luciana Almeida

Brazil

Kristian Almstrup

Denmark

Sanjeev Banerjee

United States of America

Anthony Bench

United Kingdom

Abdulbari Bener

Qatar

James Close

United Kingdom

Jeffrey Craig

Australia

Pierre Dehan

Belgium

David Diaz-Sanchez

United States of America

Christian Dransfeld

Germany

Thomas Eggermann

Germany

Alberto Ferlin

Italy

Gary Firestein

United States of America

Simonetta Friso

Italy

Daria Grafodatskaya

Canada

Joerg Gromoll

Germany

Richard Hamelin

France

Dana Hancock

United States of America

Anja Harder

Germany

Jaime Hart

United States of America

Georges Herbein

France

Anelia Horvath

United States of America

Cathrine Hoyo

United States of America

Jian Huang

China

Katsuhide Igarashi

Kenya

Riadh Jemaa

Tunisia

Nagaya Ken

Japan

Matthias Linke

Germany

Augusto Litonjua

United States of America

Amar Mehta

United States of America

Ramzi Mohammad

United States of America

Ian Morison

New Zealand

Vundavalli Murty

United States of America

Atsushi Natsume

Japan

Priscilla Negraes

Brazil

Takahiro Ochiya

Japan

Shuji Ogino

United States of America

Agnieszka pdowska

Germany

Igor Pogribny

United States of America

Jan Purrucker

Germany

M Ram Sairam

Canada

Yogen Saunthararajah

United States of America

Markus Schaich

Germany

Wei Shen

United States of America

Bente Talseth-Palmer

Australia

Mary Beth Terry

United States of America

Hwei-Fang Tien

Taiwan

Robert Wright

United States of America

Weihua Zeng

United States of America

Yujing Zhang

United States of America

Jinying Zhao

United States of America

Jingde Zhu

China

Vlatka Zoldos

Croatia

